# Simulated microgravity significantly altered metabolism in epidermal stem cells

**DOI:** 10.1007/s11626-020-00435-8

**Published:** 2020-03-20

**Authors:** Bin-Bin Li, Zheng-Yang Chen, Nan Jiang, Song Guo, Jia-Qi Yang, Shao-Bin Chai, Hong-Feng Yan, Pei-Ming Sun, Gang Hu, Tao Zhang, Bing-Xin Xu, Hong-Wei Sun, Jin-Lian Zhou, He-Ming Yang, Yan Cui

**Affiliations:** 1 Department of General Surgery, PLA 306 Clinical Hospital of Anhui Medical University, Beijing, 230000 China; 2 Department of General Surgery, PLA 306 Teaching Hospital of Peking University Health Science Center, Beijing, 100101 China; 3 The Center for Hepatopancreatobiliary Diseases, Beijing Tsinghua Changgung Hospital, Tsinghua University, Beijing, 102218 China; 4 Department of General Surgery, 306 Hospital of PLA, Beijing, 100101 China; 5 Medicine and Experimental Research Center, 306 Hospital of PLA, Beijing, 100101 China; 6 Department of Pathology, 306 Hospital of PLA, Beijing, 100101 China

**Keywords:** Epidermal stem cells, Metabolism, Simulated microgravity, RCCS, Metabolism pathway

## Abstract

Simulated microgravity can significantly affect various cell types and multiple systems of the human body, such as cardiovascular system, skeletal muscle system, and immune system, and is known to cause anemia and loss of electrolyte and fluids. Epidermal stem cells (EpSCs) were cultured in a rotary cell culture system (RCCS) bioreactor to simulate microgravity. The metabolites of EpSCs were identified by liquid chromatography-mass spectrometry (LC-MS). Compared with normal gravity (NG) group, a total of 57 different metabolites of EpSCs were identified (*P* < 0.05, VIP > 1), including lipids and lipid-like molecules (51 molecules), amino acids (5 molecules), nucleosides, nucleotides, and analogues (1 molecule). According to the partial least squares discriminant analysis (PLS-DA) score plot, a VIP > 1 and *P* < 0.05 were obtained for the 57 different metabolites, of which 23 molecules were significantly downregulated and 34 were significantly upregulated in simulated microgravity (SMG) group. These results showed that SMG has a significant impact on different pathways, and Kyoto Encyclopedia of Genes and Genomes (KEGG) pathway enrichment analysis indicated that multiple pathways were involved, mainly the amino acid metabolism pathway, lipid metabolism pathway, membrane transport pathway, and cell growth and death pathways. Thus, the metabolic profile of EpSCs was changed under SMG. Exploring the metabolic profile of EpSCs would be helpful to further understand the growth characteristics of EpSCs under SMG, which will provide a new approach to explore the metabolomics mechanism of stress injury and repair trauma under SMG.

**Electronic supplementary material:**

The online version of this article (10.1007/s11626-020-00435-8) contains supplementary material, which is available to authorized users.

## Introduction

The epidermis is the outermost layer of the skin, which forms an effective barrier protecting our body from the hostile environment and from dehydration (Moestrup *et al.*^2017^). The epidermis is a constantly renewing, stratified squamous epithelium that is mainly comprised of epidermal stem cells (EpSCs) structured as different cellular layers. EpSCs have the following general characteristics of stem cells: (1) high ability for self-renewal, (2) ability for slow cell cycle, and (3) ability to adhere to basement membranes (Sambandam *et al.*^2014^). In recent years, increasing attention has been paid to EpSCs and its role in homeostasis, wound repair, and tumorigenesis. EpSCs are also becoming increasingly attractive as an option in gene therapy, tissue bioengineering, and treatment of various other diseases (Andreadis 2007; Chebotaev *et al.*^2007^).

Rotary cell culture system (RCCS) is an instrument which can cultivate cells under simulated microgravity on the ground (Jiang *et al.*^2014^). Lei *et al.* (2011) suggested that, compared with the static culture group, the proliferation ability of EpSCs significantly changed, and cells could generate three-dimensional epidermal aggregates under feeder-free culture condition; however, the RCCS could potentially provide a good environment to inhibit the differentiation of EpSCs. In recent years, the application of metabolomics to study the concentration and flux of small molecules in cells has allowed the identification of biomarkers and study of the influence of the environment on the metabolic pathways of stem cells (Patti *et al.*^2013^). Metabolomics can also help classify cells according to their molecular characteristics, identify metabolite biomarkers in biological samples, and evaluate the effects of different drugs on cells and tissues (Panopoulos *et al.*^2012^; Armitage and Barbas 2014). Studies have made significant progress in understanding the regulation of metabolism, especially the interaction between growth control, differentiation, signaling pathways, and metabolism (Yuan *et al.*^2013^; Jung *et al.*^2018^). Furthermore, many studies have found relative availability of amino acids, nitrates, sugars, and lipid metabolism regulation as metabolic intermediates as epigenetic modification cofactors (Harvey *et al.*^2016^; Lees *et al.*^2017^). These metabolites represent the final downstream products of the genome while providing an instantaneous insight into the physiological state of the organism and its interaction with the environment (Llorach *et al.*^2012^). Metabolomics may be the ideal tool to rapidly characterize and anticipate substances within cells. However, metabolism is affected by various environmental factors, such as hypoxia and microgravity (Meijer *et al.*^2012^). Moreover, homeostasis is maintained by diverse cells under widely varying environments realized through interactions between metabolic networks, allosteric regulation, signaling pathways, and transcription factors (Mulukutla *et al.*^2016^). Many studies have shown that metabolism plays an important role in controlling proliferation, differentiation, and migration of stem cells (Sun *et al.*^2019^; Jeong *et al.*^2019^). However, there have been no reports on the metabolism of EpSCs under microgravity. The aim of the present study was to investigate the effects of simulated microgravity on metabolism of EpSCs. The significance of this study is to further understand how microgravity induces the metabolic changes in EpSCs and to establish the foundation for further studies on EpSCs.

## Materials and Methods

### Experimental groups and culture of EpSCs

EpSCs were purchased from Beijing Bei Na Chuang Lian Biotechnology Research Institute (Cell identification is shown in Supplementary 1). EpSCs was cultured in DMEM (Thermo Fisher Scientific, Waltham, MA) and divided into the simulated microgravity (SMG) group and normal gravity (NG) group (1 g static cultures). Simulated microgravity conditions were simulated using an RCCS (Facer *et al.*^2005^). The cultured solution is composed of 94% high-glucose medium (Sigma, Burlington, MA), 5% fetal bovine serum (Sigma), and 1% penicillin-streptomycin (100 units/ml penicillin and 100 mg/ml streptomycin, Thermo Fisher Scientific, Waltham, MA). The SMG group and NG group were maintained at 37°C in a humidified 5% CO_2_ incubator. EpSCs were observed every day, and fresh medium was supplemented every 2 to 3 d.

### Microcarrier preparation, seeding, and culture

In our study, cytodex-3 microcarriers (Sigma) were carriers used in the SMG group. The cytodex-3 microcarriers were immersed in 0.1 mol/l phosphate-buffered saline (PBS) and left in the refrigerator at 4°C for at least 4 h; then, it was washed with alcohol 3 times and placed in a 4°C refrigerator overnight. When cytodex-3 microcarriers were used, the carriers were rinsed with 0.1 mol/l PBS for 3 times. For culture, 0.5 × 10^6^ cells/ml EpSCs and 5 mg/ml microcarriers were inoculated to 10-ml culture vessel of RCCS. Along with this, the 5-ml syringe was connected to the liquid channel for exhaust, and then, high-aspect rotating vessel (HARV) was connected to RCCS after confirming that the bubbles were removed. The SMG group HARV axis were parallel to the ground and the NG group HARV axis were perpendicular to the ground. Twelve samples were studied at each time point resulting in a total of 24 samples. The initial mediation speed of RCCS is 10 rpm/min, which was rotated once every 5 min and then stopped. After resting for 1 min, it was repeated 3 times. After the mediation, the rotating speed was carried out as described by Lei *et al.*, a low level of about 12 rpm/min on the first day. On the second day, the rotating speed was increased to 22 rpm/min until the end of 3-d culture (Lei *et al.*^2011^). The RCCS was observed, and its speed was adjusted constantly to ensure that the microcarriers are always suspended in the culture medium. Experiments were performed for 1 d and 3 d.

### Sample collection and processing

The HARV solution was extracted with a 5-ml syringe and placed in a 15-ml centrifuge tube. The solution was stationary for several minutes, and the supernatant was discarded after the complex precipitation of the cell microcarriers. An appropriate amount of 0.1 mol/l PBS was used to wash the cell microcarrier complex, which was repeated 3 times and the supernatant was discarded. Then, at 37°C, 2–3 ml trypsin (0.25% EDTA) was added to the centrifuge tube. After 4–5 min of digestion and intermittent percussion to complete the digestion, the reaction was terminated. Medium was added to the centrifuge tube and stirred carefully with the straw to make the cells on the microcarrier fall off. Then, the suspension was centrifuged at 1000 rpm/min for 5 min. After centrifugation, the single-cell suspension was obtained by means of microcarrier 70-μm cell sieve. The NG group cells were washed 2 times by 0.1 mol/l PBS, then digested with 2–3 ml trypsin (0.25% EDTA) at 37°C for 5 min. The digestion was terminated by adding DMEM. Then, the suspension was centrifuged at 1000 rpm/min for 5 min. The unicellular suspensions of the NG and SMG group cells were frozen in liquid nitrogen for a few minutes, and then stored in a − 80°C freezer.

The appropriate amount of cell samples from the NG group and SMG group was added with 400 μl cold methanol in the sample, which was broken by a high-throughput tissue fragmentation apparatus at low temperature. Then, 100 μl distilled water was added to the samples, and the samples were mixed with vortex. Finally, ultrasonic extraction was performed on ice for 10 min, and these steps were repeated for 3 times. In the last step, the sample was prepared at − 20°C for 30 min and centrifuged at 13,000 rpm, at 4°C for 15 min; then, the supernatant was discarded and drained. Finally, 100 μl of acetonitrile:water (1:1) solution was added into the tube for testing.

### LC-MS metabolite analysis

The platform of the ultraperformance liquid chromatography-tandem mass spectrometry (UPLC-MS/MS) was based on Ultimate 3000-Velos Pro system (Waters, Milford, MA) equipped with a binary solvent delivery manager and a sample manager, and samples were analyzed using LTQ Orbitrap Mass Spectrometer equipped with an electrospray interface analyzer (Thermo Fisher Scientific). The samples were redissolved in precooled methanol:water (v:v, 7:3)and the injection volume was fixed at 180 μl. One of the compounds was analyzed by acid positive ion optimization and the other was analyzed using basic negative ion-optimized conditions with two independent injections using separate dedicated ACQUITY BEH C18 column (100 mm × 2.1 mm i.d., 1.7 μm; Waters). The mobile phase was at a flow rate of 0.40 ml/min, where B was acetonitrile (0.1% (v/v) formic acid) and A was aqueous formic acid (0.1% (v/v) formic acid) in positive mode, and B is acetonitrile (containing 5 mM ammonium formate) and A is water (containing 5 mM Ammonium formate) in negative mode. The injection volume was 3.0 μl. The column was maintained at 45°C. The MS data was collected using LTQ Orbitrap mass spectrometer equipped with an electrospray ionization (ESI) source operating in either positive or negative ion modes. The capillary and source temperature were set at 350°C, and the dissolved gas flow rate was 45 l/h. The scan range was 50**~**1000 mass-to-charge ratios (m/z), and the resolution was 30,000. Nitrogen was used for all types of gases. Data are represented as the mean ± SEM from 6 repeated experiments.

### Data processing and statistical analysis

UHPLC-LTQ-MS data were processed using progenesis QI (Waters Corporation, Milford, MA). Metabolites were searched using the Human Metabolome Database (HMDB, http://www.hmdb.ca) or Kyoto Encyclopedia of Genes and Genomes (KEGG, http://www.genome.jp). Before statistical analysis, the result data sets of the positive and negative ion modes were merged into one data set. The selection of statistically significant metabolites was performed based on partial least square discriminant analysis (PLS-DA) models obtained using the software package SIMCA-P+14.0 (Umetrics, Umeå, Switzerland). The higher the variable importance in the projection (VIP) value, the greater the contribution to the classification. The VIP values > 1.0 were considered significant. Data were compared by 2-sample or paired Student’s *t* test, analysis of variance, or chi-square test, when appropriate. *P* < 0.05 was considered to be statistically significant.

## Results

### Comparative metabolite analysis of EpSCs between NG group and SMG group

**Figure 1.**
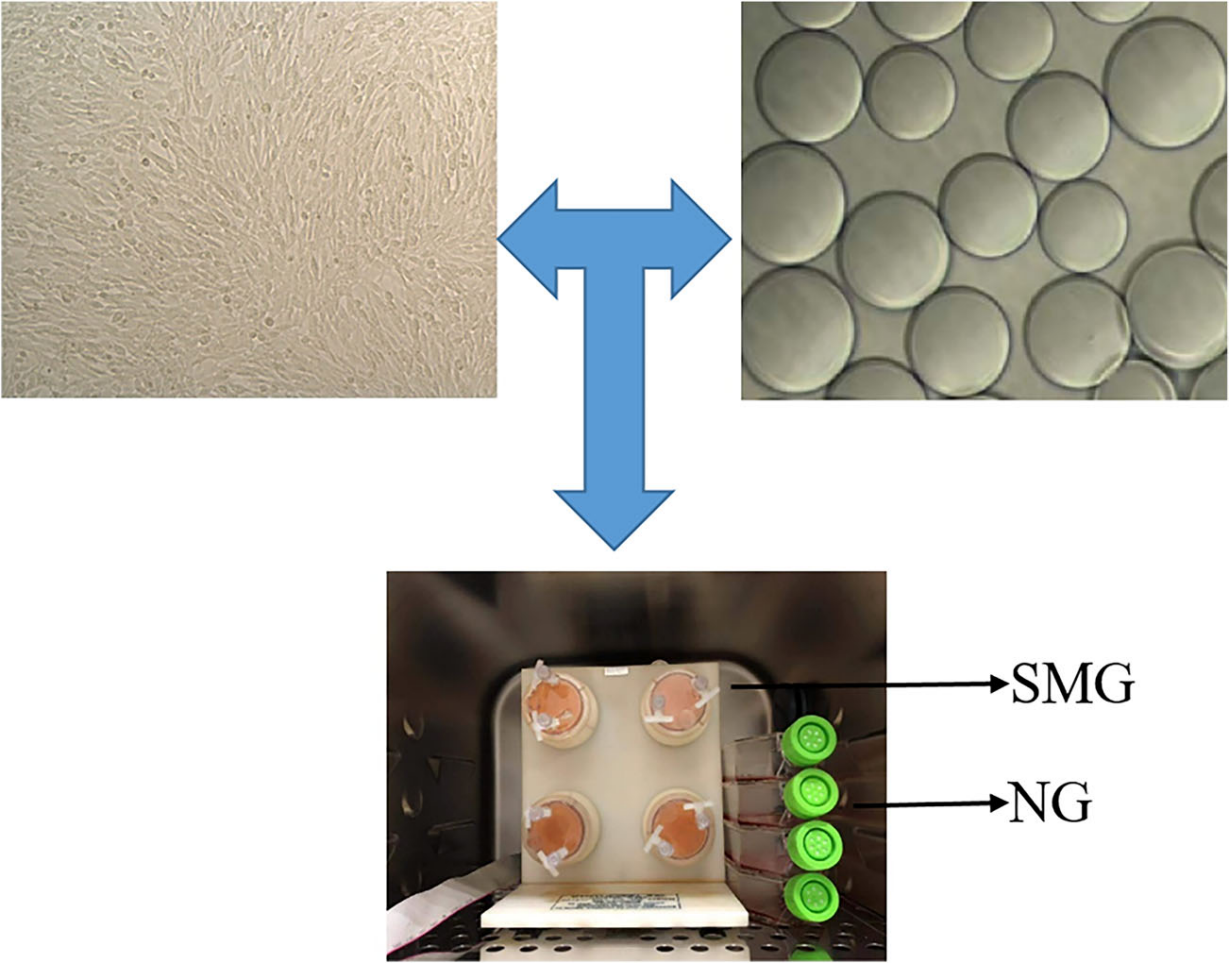
Schematic illustration of our experiment used to culture EpSCs on cytodex-3 microcarriers under normal gravity culture and RCCS. The vessel was installed onto the RCCS rotating in a clockwise direction at a speed of 12 rpm in the first d. After cells adhered to the cytodex-3 microcarriers 24 h later, the rotation speed was adjusted to 22 rpm.

**Figure 2.**
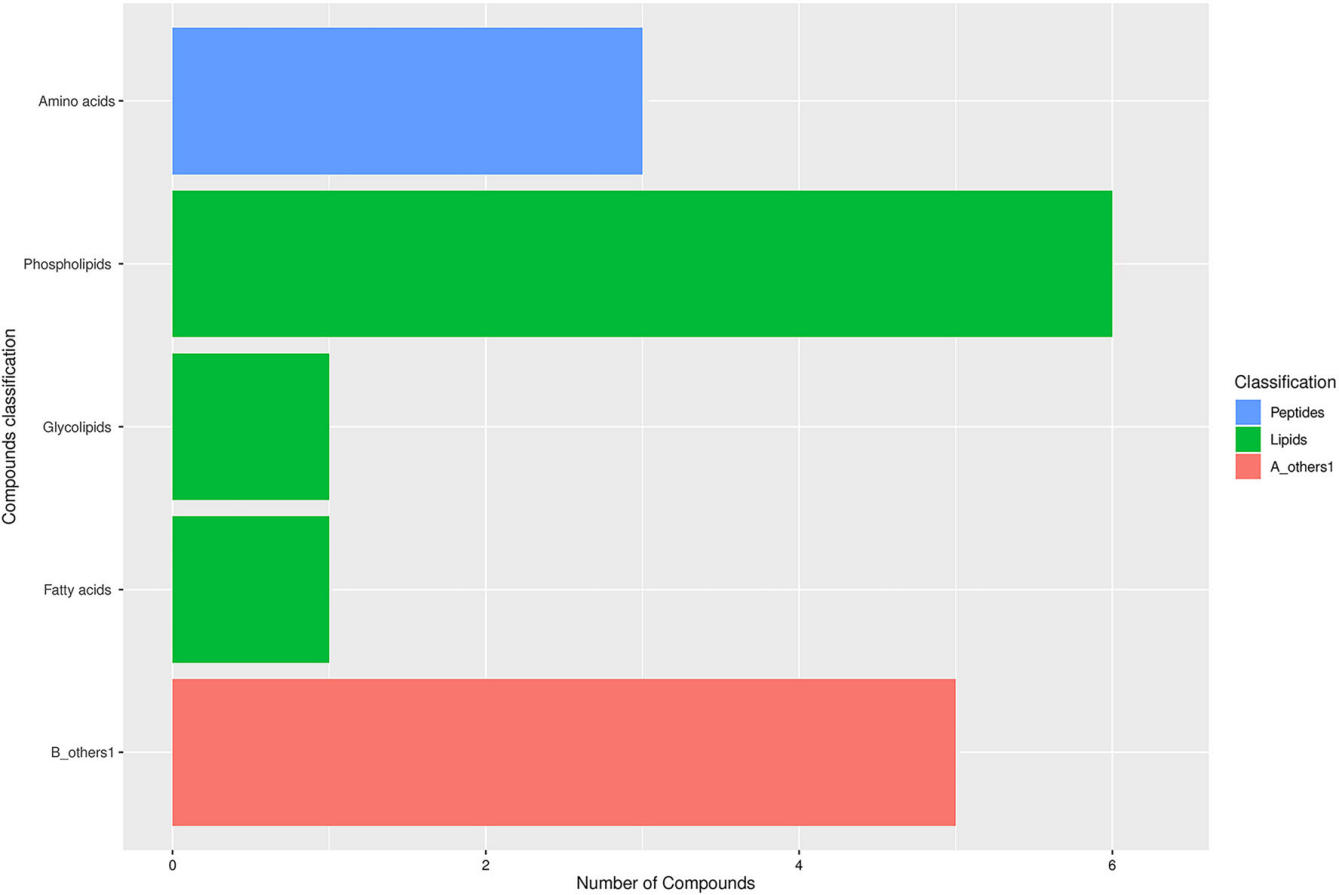
Fifty-seven differential metabolites. KEGG compound classification of 57 differential metabolites, including glycolipids, phospholipids, fatty acids, and amino acids (*P* < 0.05 and VIP > 1).

**Figure 3.**
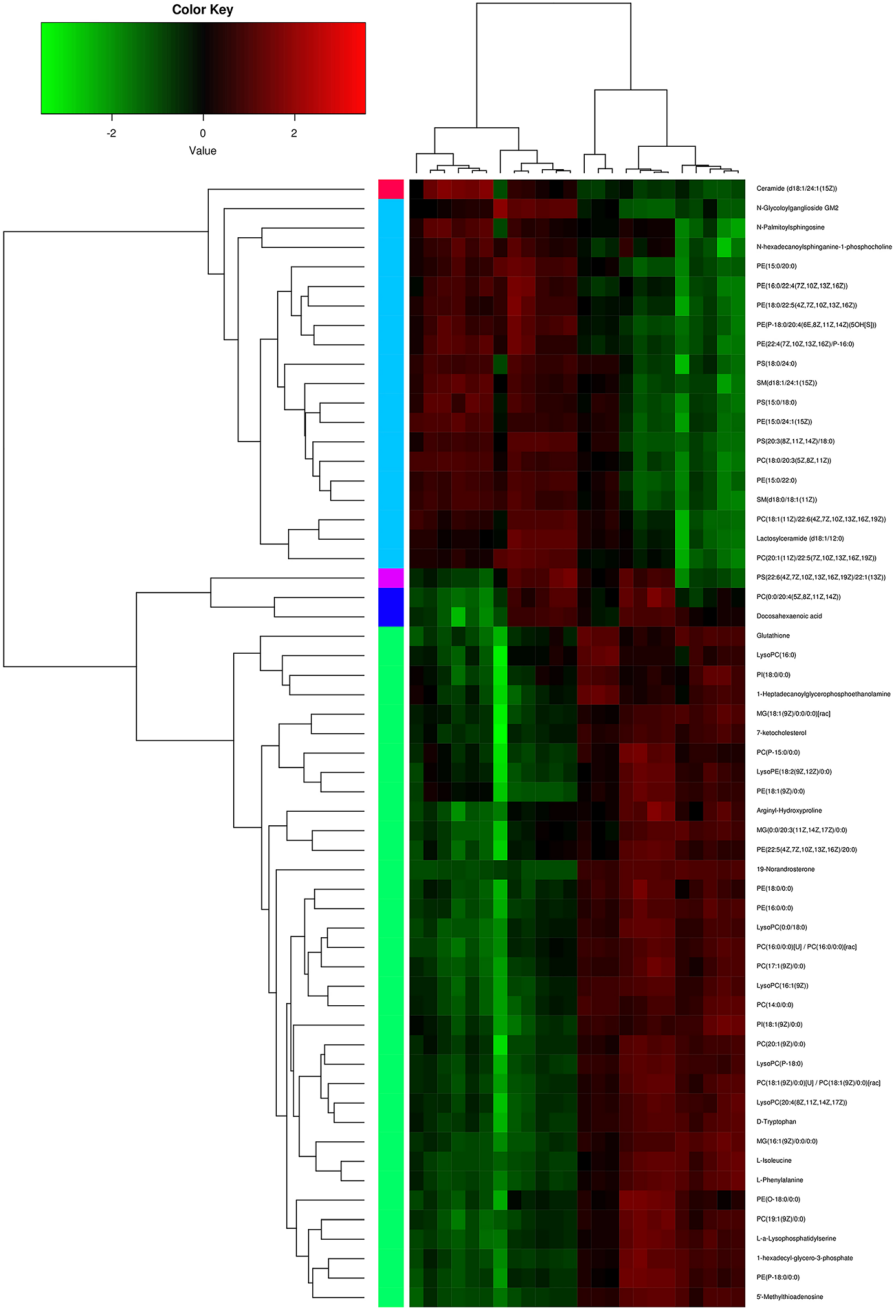
Heat map of 57 differential metabolites. The *colorkey* above the heat map indicates the expression level of these 57 differential metabolites, and *red* represents upregulation while *green* represents downregulation.

In order to study the effect of microgravity on altered metabolites of EpSCs, cells were digested by trypsin, and cytodex-3 microcarriers were inoculated in a rotary bioreactor for 1 d, which allowed cells to adhere to the surface of the beads. Then, the sample was divided into two groups, including the simulated microgravity group in the RCCS and normal gravity group in T75 cell culture flasks (Fig. 1). To clarify the effect of SMG on altered metabolites of EpSCs, two NG (N1 and N3) samples and two SMG (S1 and S3) samples were analyzed by the LC-MS/MS. Then, the KEGG and HMDBs database were searched to assess the compound classification, which led to the detection of 57 metabolites (*P* < 0.05 and VIP > 1) (Fig. 2) in 4 classes, including lipids and lipid-like molecules (51 molecules), amino acids (5 molecules), nucleosides, nucleotides, and analogues(1 molecule). According to the PLS-DA score plot, a VIP > 1 and *P* < 0.05 were obtained for 57 different metabolites; of these, 23 molecules were significantly downregulated and 34 molecules were significantly upregulated in the SMG group (all *P* < 0.05; Table 1). Compared with the NG group, a total of 57 different metabolites of EpSCs are performed with hierarchical clustering analysis, showing the variation trend of metabolite expression, and only statistically significant changes are shown (VIP > 1, *P* < 0.05) (Fig. 3).

**Table 1.** Altered metabolites with statistical significance between SMG and NG

	Metabolites	VIP score (VIP > 1.0)	*P* value
Upregulated	l-a-Lysophosphatidylserine	2.0150	< 0.05
	LysoPC(16:0)	3.97520	< 0.05
	LysoPE(18:2(9Z,12Z)/0:0)	1.20850	< 0.05
	LysoPC(20:4(8Z,11Z,14Z,17Z))	1.54970	< 0.05
	LysoPC(16:1(9Z))	6.72790	< 0.05
	LysoPC(P-18:0)	4.23040	< 0.05
	LysoPC(0:0/18:0)	6.43030	< 0.05
	PC(P-15:0/0:0)	1.60280	< 0.05
	PC(17:1(9Z)/0:0)	1.91910	< 0.05
	PC(16:0/0:0)/PC(16:0/0:0)	11.6912	< 0.05
	PC(18:1(9Z)/0:0)	13.2491	< 0.05
	PC(14:0/0:0)	2.66480	< 0.05
	PC(19:1(9Z)/0:0)	1.41640	< 0.05
	PC(20:1(9Z)/0:0)	2.76320	< 0.05
	PE(18:1(9Z)/0:0)	5.18730	< 0.05
	PE(18:0/0:0)	2.84110	< 0.05
	PE(22:5(4Z,7Z,10Z,13Z,16Z)/20:0)	1.43070	< 0.05
	PE(16:0/0:0)	2.01650	< 0.05
	PE(O-18:0/0:0)	1.83920	< 0.05
	PE(P-18:0/0:0)	2.87990	< 0.05
	PI(18:0/0:0)	2.08050	< 0.05
	PI(18:1(9Z)/0:0)	1.80560	< 0.05
	1-Hexadecyl-glycero-3-phosphate	2.65970	< 0.05
	MG(0:0/20:3(11Z,14Z,17Z)/0:0)	1.45120	< 0.05
	MG(16:1(9Z)/0:0/0:0)	1.97220	< 0.05
	MG(18:1(9Z)/0:0/0:0)	3.76530	< 0.05
	19-Norandrosterone	1.46090	< 0.05
	7-Ketocholesterol	1.25320	< 0.05
	d-Tryptophan	1.63380	< 0.05
	Glutathione	3.17470	< 0.05
	l-Isoleucine	1.63560	< 0.05
	l-Phenylalanine	2.18430	< 0.05
	Arginyl-hydroxyproline	1.36820	< 0.05
	5′-Methylthioadenosine	3.57950	< 0.05
Downregulated	PC(18:1(11Z)/22:6(4Z,7Z,10Z,13Z,16Z,19Z))	2.85060	< 0.05
	PC(20:1(11Z)/22:5(7Z,10Z,13Z,16Z,19Z))	1.90810	< 0.05
	PC(0:0/20:4(5Z,8+A28:F38Z,11Z,14Z))	1.00600	< 0.05
	PC(18:0/20:3(5Z,8Z,11Z))	1.34610	< 0.05
	PE(15:0/22:0)	1.61560	< 0.05
	PE(22:4(7Z,10Z,13Z,16Z)/P-16:0)	3.63830	< 0.05
	PE(P-18:0/20:4(6E,8Z,11Z,14Z)(5OH[S]))	3.50460	< 0.05
	PE(18:0/22:5(4Z,7Z,10Z,13Z,16Z))	1.57940	< 0.05
	PE(15:0/20:0)	3.08910	< 0.05
	PE(15:0/24:1(15Z))	4.63110	< 0.05
	PE(16:0/22:4(7Z,10Z,13Z,16Z))	3.81120	< 0.05
	PS(15:0/18:0)	1.76470	< 0.05
	PS(18:0/24:0)	2.09720	< 0.05
	PS(20:3(8Z,11Z,14Z)/18:0)	2.67050	< 0.05
	PS(22:6(4Z,7Z,10Z,13Z,16Z,19Z)/22:1(13Z))	1.06960	< 0.05
	Docosahexaenoic acid	1.97200	< 0.05
	Ceramide (d18:1/24:1(15Z))	1.80790	< 0.05
	N-Glycoloylganglioside GM2	2.12900	< 0.05
	N-Palmitoylsphingosine	1.60520	< 0.05
	Lactosylceramide (d18:1/12:0)	2.95220	< 0.05
	SM(d18:0/18:1(11Z))	1.43280	< 0.05
	SM(d18:1/24:1(15Z))	3.18440	< 0.05
	N-Hexadecanoylsphinganine-1-phosphocholine	1.46990	< 0.05

*VIP*, variable importance in the projection

### Lipids and lipid-like metabolite alterations of EpSCs between NG group and SMG group

As described in the above section, compared with the NG group, 51 molecules of lipids and lipid molecules were detected by m/z search for the characteristics of the identified metabolites that are significantly different between the NG group and SMG group by the KEGG database and Human Metabolome Database (HMDB) (Table 2). These differential metabolites, which satisfied the requirements of a VIP > 1, and *P* < 0.05 according to Student’s *t* test, which contributed to the characterization of differences between NG and SMG cells, included phospholipids (31 molecules), sphingolipids (7 molecules), glycerolipids (3 molecules), lysophospholipids (7 molecules), steroids and steroid derivatives (2 molecules), and fatty acyls (1 molecule) (Table 3). For all lipid classes, the masses of the lipid species altered in the 1- and 3-d SMG group were much greater than those in the 1- and 3-d NG group. Among the 31 phospholipid metabolites, 15 molecules of metabolites were significantly downregulated, including 6 molecules of phosphatidylethanolamine (PE), 4 molecules of phosphatidylserine (PS), 2 molecules of PI, and 4 molecules of phosphatidylcholine (PC). Fifteen molecules of metabolites were significantly downregulated, including 7 molecules of PC, 4 molecules of PS, and 4 molecules of PC. Seven molecules of sphingolipid metabolites were significantly decreased. Seven molecules of lysophospholipids were significantly upregulated. Two molecules of steroids and steroid derivatives were significantly upregulated. All the 3 molecules glycerolipid metabolites were significantly upregulated.

**Table 2.** Fifty-one lipids and lipid molecules

Lipid category species	Lipid class	Abbreviation	No. of lipid
Phospholipid	Phosphatidylcholine	PC	10
	Alkenylphosphatidylcholine	PC(P)	1
	Phosphatidylethanolamine	PE	10
	Alkylphosphatidylethanolamine	PE(O)	1
	Lkylphosphatidylethanolamin	PE(P)	2
	Phosphatidylinositol	PI	2
	Phosphatidylserine	PS	4
	1-Hexadecyl-glycero-3-phosphate	PA	1
Sphingolipid	Sphingomyelin	SM	3
	Ceramide	Cer	2
	Glycosphingolipid	Gcer	1
	Lactosylceramide	Lcer	1
Glycerolipide	Monoacylglycerol	MG	3
Lysophospholipid	Lysophosphatidylethanolamine	LPE	1
	Lyso-phosphatidylcholine	LPC	5
	l-a-Lysophosphatidylserine		1
Fatty acyls	Docosahexaenoic acid	DHA	1
Steroids	19-Norandrosterone		1
Steroid derivatives	7-Ketocholesterol		1
Total lipids			51

VIP > 1 and *P* < 0.05

**Table 3.** Fifty-one specific lipids and lipid molecules

PC	PC(18:1(11Z)/22:6(4Z,7Z,10Z,13Z,16Z,19Z))
	PC(20:1(11Z)/22:5(7Z,10Z,13Z,16Z,19Z))
	PC(0:0/20:4(5Z,8+A28:F38Z,11Z,14Z))
	PC(18:0/20:3(5Z,8Z,11Z))
	PC(P-15:0/0:0)
	PC(17:1(9Z)/0:0)
	PC(16:0/0:0)
	PC(18:1(9Z)/0:0)
	PC(14:0/0:0)
	PC(19:1(9Z)/0:0)
	PC(20:1(9Z)/0:0)
PE	PE(15:0/22:0)
	PE(22:4(7Z,10Z,13Z,16Z)/P-16:0)
	PE(P-18:0/20:4(6E,8Z,11Z,14Z)(5OH[S]))
	PE(18:0/22:5(4Z,7Z,10Z,13Z,16Z))
	PE(15:0/20:0)
	PE(15:0/24:1(15Z))
	PE(16:0/22:4(7Z,10Z,13Z,16Z))
	PE(18:1(9Z)/0:0)
	PE(18:0/0:0)
	PE(22:5(4Z,7Z,10Z,13Z,16Z)/20:0)
	PE(16:0/0:0)
	PE(O-18:0/0:0)
	PE(P-18:0/0:0)
PI	PI(18:0/0:0)
	PI(18:1(9Z)/0:0)
PS	PS(15:0/18:0)
	PS(18:0/24:0)
	PS(20:3(8Z,11Z,14Z)/18:0)
	PS(22:6(4Z,7Z,10Z,13Z,16Z,19Z)/22:1(13Z))
PA	1-Hexadecyl-glycero-3-phosphate
SM	SM(d18:0/18:1(11Z))
	SM(d18:1/24:1(15Z))
	N-Hexadecanoylsphinganine-1-phosphocholine
Lcer	Lactosylceramide (d18:1/12:0)
Cer	Ceramide (d18:1/24:1(15Z))
	N-Palmitoylsphingosine
Gcer	N-Glycoloylganglioside GM2
LPC	LysoPC(16:0)
	LysoPC(20:4(8Z,11Z,14Z,17Z))
	LysoPC(16:1(9Z))
	LysoPC(P-18:0)
	LysoPC(0:0/18:0)
LPE	LysoPE(18:2(9Z,12Z)/0:0)
	l-a-Lysophosphatidylserine
MG	MG(0:0/20:3(11Z,14Z,17Z)/0:0)
	MG(16:1(9Z)/0:0/0:0)
	MG(18:1(9Z)/0:0/0:0)
Steroids	19-Norandrosterone
Steroid derivatives	7-Ketocholesterol C27H44O2
Fatty acyls	Docosahexaenoic acid

### KEGG pathway enrichment analysis

The KEGG pathway was divided into seven categories: metabolism, genetic information processing, environmental information processing, cellular processes, organismal systems, human diseases, and drug development.

**Figure 4.**
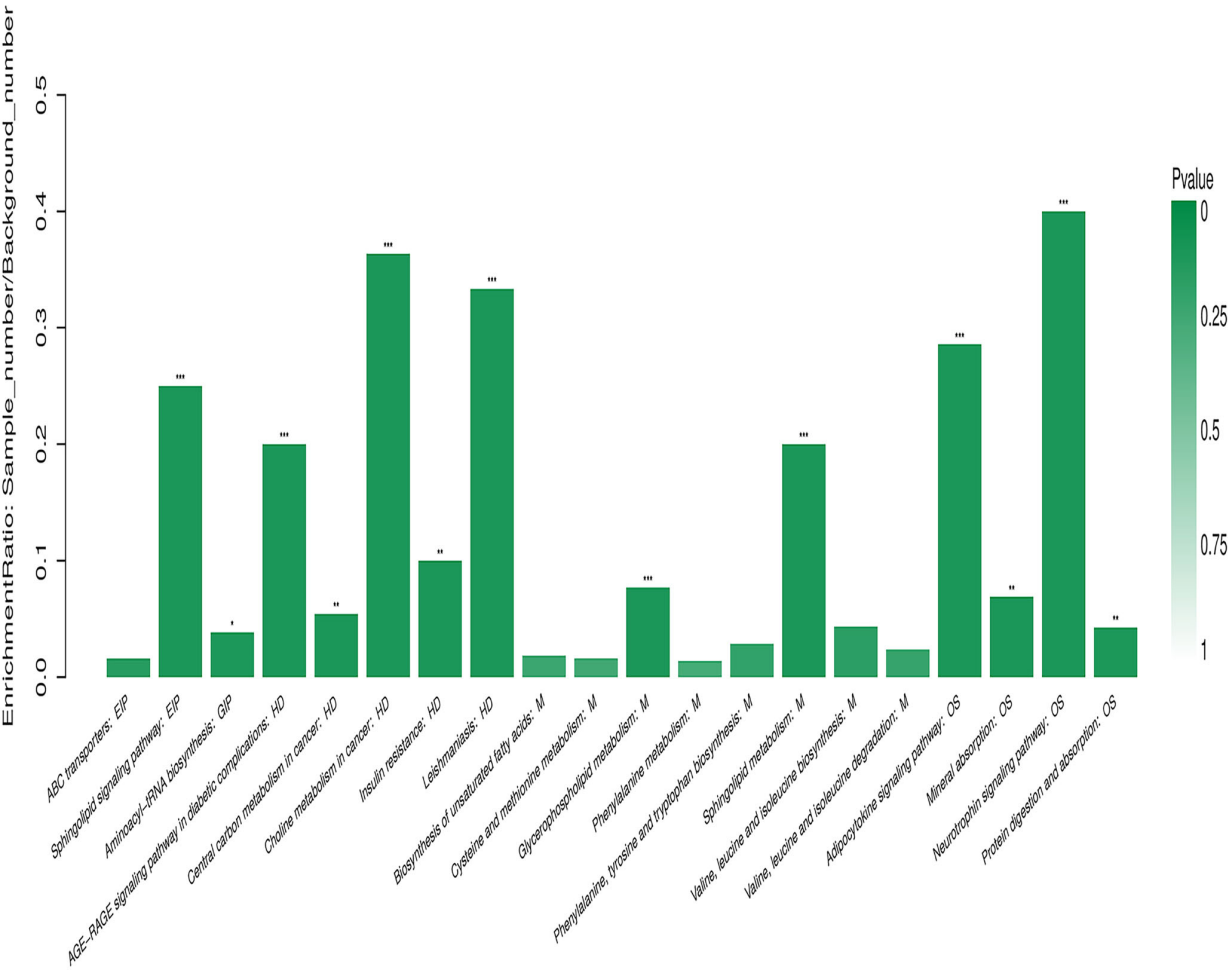
KEGG pathway enrichment analysis. The abscissa represents pathway name, and the ordinate represents enrichment rate, indicating the ratio of the number of metabolites enriched in the pathway to the number of metabolites annotated to the pathway. The larger the ratio, the greater the enrichment degree. *Column color gradient* indicates the significance of enrichment. The darker the default color is, the more significant the KEGG term is. *P* value or FDR < 0.001 is marked with ***, *P* value or FDR < 0.01 is marked with **, and *P* value or FDR < 0.05 is marked with *.

Our results showed that SMG can have a significant impact in different pathways, and the KEGG pathway enrichment analysis indicated that the multiple pathways were mainly involved, including amino acid metabolism pathway, lipid metabolism pathway, membrane transport pathway, and neurotrophin signaling pathway (Fig. 4), indicating its role in cell growth and death pathway.

## Discussion

The complex structure of the skin is comprised of cells from different lineages. The structure is maintained mainly through stem cells in a structure called “niches” in the skin, which creates a suitable microenvironment for them to maintain their specific characteristics (Abbas and Mahalingam 2009). EpSCs, which play an important role in the renewal and repair of epidermis, are considered to have a potentially unlimited ability to divide and differentiate into multiple target cells. However, many factors are likely to adversely affect the growth and metabolism of EpSCs, such as microgravity and ultraviolet radiation. More importantly, microgravity, as a special stressor, can affect cell metabolism, morphology, proliferation, cell cycle distribution, and apoptosis (Sokolovskaya *et al.*^2014^; Zhu *et al.*^2014^; Chen *et al.*^2019^). In our study, we explored whether 1- and 3-d exposure to microgravity could change the metabolites of EpSCs. In order to achieve this, we performed liquid chromatography-mass spectrometry (LC-MS) is used to assess the relative trends of the metabolites in different samples, especially those involved in lipid metabolism. The significant different metabolites between the NG group and SMG group were searched against the KEGG database and the HMDB with the obtained m/z data. To narrow down to the metabolites of interest, which satisfied the requirements of a VIP > 1, *P* < 0.05 according to Student’s *t* test was adopted to identify metabolites that were differentially expressed. Our study revealed 57 distinct molecules of metabolites of EpSCs (as seen in Tables 1 and 2) when the cells were subjected in the 1- and 3-d SMG.

Cell membranes are comprised of lipids and proteins. The lipids in the bilayer are mainly comprised of phospholipids and glycosphingolipids. For phospholipids, they mainly consist of phosphatidylcholine (PC), phosphatidylethanolamine (PE), phosphatidylserine (PS), and sphingomyelin (SM), which are the backbone of most biofilms. In addition, a common feature of mammalian cell membranes is the asymmetric distribution of phospholipids between bilayer lobules, which facilitates the function of phospholipids in some cellular processes (Umeda and Emoto 1999; Vance 2013). Lipids are not only components of the cell membrane but also play an important role in cell proliferation and apoptosis. The study had found that when the content of PE in mitochondria (mtPE) is reduced, it affects cell growth leading to reduced ATP production and a decreased respiratory capacity of the cell. In addition, the ultrastructure of mitochondria was seriously aberrant, and mitochondria were widely fragmented (Tasseva *et al.*^2013^). Other studies have shown that when cell-surface PE was specifically bound to the peptide and immobilized, actin filaments disintegrated at the cleavage furrow and subsequent membrane fusion and cell division were blocked, suggesting that PE was involved in the recombination of actin filaments in the process of apoptosis. In addition, when the PE level decreased by 50%, cell division was inhibited in mutant CHO-K1 cells. While appropriate amounts of PE or ethanolamine were added to these cells, cell division returned to normal (Emoto *et al.*^1996^; Emoto and Umeda 2000). Therefore, we could hypothesize that PE is a promising target to detect cellular energy metabolism, cell death, and cell division. In addition, many studies found that PS, which plays an important role in mitochondrial function, is also an important precursor of mitochondrial PE. In mammalian cells, PS exists in the form of anionic phospholipids in eukaryotic biomembranes. In addition to being a structural component of the membrane, PS exposure on the cell surface is considered to be the signal for apoptotic cell recognition and phagocytosis. When PS is exposed to the outer plasma membrane of apoptotic cells, it is recognized and bound by surface receptors of macrophages. So, the expression of PS at the cell surface is one of the most important “eat me” triggers of apoptosis and phagocytosis (Fadok *et al.*^2000^; Hamon *et al.*^2000^; Vance 2013). This is consistent with the viewpoint of Yu *et al.* (2000). At the same time, PE and PC metabolize diacylglycerol, fatty acid, and phospholipid acid as second messengers, which play important roles in the cell signal conduction process (Momchilova and Markovska 1999). Jung *et al.* (2018) found that, *in vivo* experiments, when PC was injected twice into subcutaneous adipose tissue in mice, fed a high-fat diet for 8 weeks, it induced adiposysis and apoptosis in a dose-dependent manner in fully differentiated 3T3-L1 cells. PC-induced lipolysis and apoptosis in adipocytes could be related to TNFα-dependent pathways. Thus, we could hypothesize that PE, PS, and PC were involved in cell proliferation, apoptosis, and energy metabolism through different pathways. In our study, compared with those in the NG group, we have found that the level of 31 phospholipid metabolites of EpSCs changed significantly in the SMG group. Among them, 16 molecules were significantly upregulated and 15 molecules of metabolites were significantly downregulated. At the same time, we found that PCs, such as PC(P-15:0/0:0), PC(17:1(9Z)/0:0), PC(16:0/0:0)/PC(16:0/0:0), PC(18:1(9Z)/0:0), PC(18:1(9Z)/0:0), PC(14:0/0:0), PC(19:1(9Z)/0:0), and PC(20:1(9Z)/0:0), were upregulated in the SMG group, indicating that the proliferation of EpSCs was weakened and apoptosis of EpSCs was increased. But the other PEs, such as PE(18:1(9Z)/0:0), PE(18:0/0:0), PE(22:5(4Z,7Z,10Z,13Z,16Z)/20:0), PE(16:0/0:0), PE(O-18:0/0:0), and PE(P-18:0/0:0) were upregulated. At the same time, other PSs, such as PS(15:0/18:0), PS(18:0/24:0), PS(20:3(8Z,11Z,14Z)/18:0), and PS(22:6(4Z,7Z,10Z,13Z,16Z,19Z)/22:1(13Z)), and other PCs, such as, PC(18:1(11Z)/22:6(4Z,7Z,10Z,13Z,16Z,19Z)), PC(20:1(11Z)/22:5(7Z,10Z,13Z,16Z,19Z)), PC(0:0/20:4(5Z,8+A28:F38Z,11Z,14Z), and PC(18:0/20:3(5Z,8Z,11Z)), were significantly downregulated. These changes of metabolites may be associated with cell gradually adapted to the microgravity environment, but its specific mechanism needs further study.

In addition to phospholipids, sphingolipids (SPL) also play an important role in some cellular activities. Several SPL metabolites, especially ceramide (Cer) and sphingomyelin (SM), act as key bioactive molecules to control critical cellular functions, such as cell cycle, aging, apoptosis, cell migration, and inflammation (Hannun and Obeid 2008). Cerbón *et al.* (2018) reported that, myriocin, an inhibitor of sphingolipid synthesis, is used during estrus, before the start of the new cell cycle in epithelial cells. The results showed that the abundance of sphingomyelin was reduced, accompanied by the stagnation of the proliferation of uterine epithelial cells on metestrus day. Sphingolipid synthesis and signaling were involved in the proliferation of uterine epithelial cells during the estrous cycle in rats. This may be related to the PKC-AKT-GSK3b-Cyclin D3 pathway. In addition, the study found that SM is consisted of ceramides and is one of the most abundant biological sphingomyelins in eukaryotes, which constitutes one of the major components of the cell membrane (Bienias *et al.*^2016^). Asano *et al.* (2012) studied that CXCL12 was significantly inhibited upon transfection with the SMS1 or SMS2 gene or when they were treated with exogenous sphingolipid, but not when cells were treated with SMS substrate ceramide. Thus, authors hypothesized that SMS-generated SM participates in cell migration regulation through the CXCL12/CXCR4 pathway. Toshima *et al.* (2019) used SM synthase 1 (SMS1)–deficient (SMS1^−/−^) mice, which displayed low SM expression, and related TCR signaling to study the role of SM in thymocyte development. The results showed that the number of SM synthase 1 (SMS1)–deficient (SMS1^−/−^) cells decreased, while that of normal cells increased. This may be related to the ability of sphingomyelin to modulate thymocyte development by inhibiting TCR-induced apoptosis. In addition, the content of SM in cells is strictly regulated by the enzymes of SM metabolism, and the SMS1 activity establishes a balance between the synthesis and degradation of SM. However, SM hydrolyzed by SMases can increase the concentration of ceramide (Cer), which is a biologically active molecule involved in cell proliferation, growth, and apoptosis (Bienias *et al.*^2016^). Many studies have also found that ceramides play a role not only in proliferation and apoptosis in normal cells but also in cancer cells. Cer induces apoptosis in many ways; ceramide can act as a second messenger, or as a signaling lipid. Moreover, the exosomal secretion pathway of ceramide has been shown to affect the apoptosis of cancer cells (Cheng *et al.*^2017^). In addition, Cer is a precursor of many complex sphingolipids, including glycolipids, sphingomyelin, and ceramide-1-phosphate (Hannun and Obeid 2008; Gault *et al.*^2010^; Merrill Jr 2011). Meanwhile, Cer is also the key molecule in the biosynthesis of sphingolipid and ganglioside. From the above discussion, we found that ceramide and SM played an important role in cell proliferation and apoptosis. In our study, SM levels (SM(d18:0/18:1(11Z), SM(d18:1/24:1(15Z), N-hexadecanoylsphinganine-1-phosphocholine, and SM(18:0/16:0) were significantly upregulated in the SMG group. At the same time, Cer levels were also significantly decreased in the SMG group compared with those in the NG group, Furthermore, the levels of the downstream metabolites of Cer, including LacCer, were markedly decreased in EpSCs. Therefore, our metabolic results suggest that microgravity has a significant impact on the proliferation, migration, and apoptosis of cells in the context of phospholipid and sphingolipid metabolism.

Microgravity affects not only the metabolism of phospholipids and sphingolipids of EpSCs but also the metabolism of lysolecithin. Studies had found that lysophosphatidylcholine (lysoPC) is the major component of ox-LDL. In some cases, lysoPC-induced apoptosis of vascular endothelial cells and human coronary artery smooth muscle cells (SMCs). This may be associated with the involvement of TRPC1/TRPC3 channels in mediating lysopc-induced Ca^2+^ influx and apoptosis by increasing the levels of the pro-apoptosis kinases Bax and cleaved caspase-3 and inhibiting the anti-apoptosis kinase Bcl-2 and the surviving kinase p-Akt in human coronary artery SMCs (Dohi *et al.*^2013^; Wang and Li 2016). In addition, ERK activity is essential for fibroblast growth factor-2 (FGF-2)–induced endothelial cell (EC) migration and proliferation. Additionally, LysoPC-mediated inhibition of the Ras/ERK pathway contributes to the reduction of EC migration and proliferation (Rikitake *et al.*^2000^). LysoPC regulates COX-2 expression and PG production, and regulates cell proliferation, through the p38-MAPK-mediated signaling pathway in rat VSMCs (Yamakawa *et al.*^2008^). Furthermore, lysoPE also participates in the regulation of intracellular Ca^2+^, which plays an important role in cell signaling (Lee *et al.*^2015^). In our study, 7 molecules of lysophospholipids were significantly upregulated in the SMG group compared with those in the NG group, suggesting that microgravity can affect cell proliferation and migration by regulating the metabolism of lysolecithin.

In addition, our studies showed that microgravity affects not only cell metabolism but also some cellular pathways. Pathway map analysis using KEGG also revealed that multiple pathways were involved under SMG (as seen in Fig. 4). Therefore, the next step we need to explore clearly is the mechanism of the effect of SMG on these functions of EpSCs. Among them, the differentially expressed metabolites were mainly related to neurotrophin signaling pathway under microgravity. Neurotrophic factors (NTs) are members of the neuronal growth factor protein family, including nerve growth factor (NGF), brain-derived neurotrophic factor (BDNF), NT3, and NT4. More significantly, NTs, proNTs, and their receptors played an important role in stem cell proliferation and differentiation, survival, plasticity, and migration. In addition, NTs perform their biological functions mainly through two types of membrane receptors: Trk tyrosine kinase receptor and neurotrophic protein receptor p75 NTR (Mowla *et al.*^2001^; Reichardt 2006; Edelmann *et al.*^2015^). Yao *et al.* (2017) proved that exposure to NT3 increased aortic valve mesenchymal cell proliferation capacity, upregulated collagen and matrix metalloproteinase (MMP) production, and increased collagen deposition. Further research found that the above changes could be eliminated by inhibiting TRK receptors. Moreover, NT3 induced Akt phosphorylation and increased cyclin D1 level in a TRK receptor–dependent manner, which promoted cell proliferation, MMP-9, and collagen production, as well as collagen deposition. This occurs through the Trk-Akt-cyclin D1 cascade reaction. Liu *et al.* (2019) studied the role of p75NTR in NGF-induced myofibroblast differentiation and collagen synthesis by lentivirus transfection. The results further suggested that p75 NTR is preferentially expressed and sufficient to induce actin cytoskeletal remodeling, which is necessary for NGF-induced α-smooth muscle actin (α-SMA) expression. In addition, p75 NTR can also induce nuclear translocation of MRTF-A by regulating NGF, and this also contributes to the expression of α-SMA and collagen-I in myofibroblasts. This may be related to the fact that p75 NTR regulates NGF-induced myofibroblast differentiation and collagen synthesis through MRTF-A. CD271 is a neurotrophic factor (NT) receptor. NGF can also regulate the proliferation, migration, and differentiation of EpSCs by regulating the balance between CD271 and TrkA (Zhang *et al.*2018a, b). As discussed above, we found that the neurotrophin signaling pathway plays an important role in the process of cell proliferation, differentiation, and migration, and in the cytoskeleton.

According to the discussion described above, EpSCs were highly sensitive to SMG, and SMG is of great value for cell research. The ground-based microgravity simulators used in our research, such as the RCCS, greatly enhance our understanding of how SMG affects humans and other organisms. Though this is the first study of SMG-induced metabolic changes in EpSCs, our understanding is far from comprehensive. But our results will help us to further study how cells regulate their physiological properties through metabolism under microgravity.

## Conclusion

In conclusion, our results of this study demonstrate 1- and 3-day exposure to SMG could change the metabolites of EpSCs, especially lipids and amino acids. The changes of these metabolites not only affect cells proliferation, differentiation, and apoptosis, but also affect cell morphology. This provides the next step to continue to study the mechanism of microgravity on cells.

## Electronic supplementary material

ESM 1 (PDF 165 kb)
