# Supplementary material for: Simulated microgravity significantly altered metabolism in epidermal stem cells
Source: In Vitro Cell Dev Biol Anim. 2020 Mar 20;56(3):200–12. doi: 10.1007/s11626-020-00435-8 (PMC7186248; doi:10.1007/s11626-020-00435-8)
Supplement: Supplementary file 1 — (PDF 165 kb) [file 11626_2020_435_MOESM1_ESM.pdf]

Report of Cell Line STR（short tandem repeat）

Experimental method：Extract DNA from samples with genome extraction kit（Axygen）.Amplificat DNA（locus in human DNA :D4S2408）

Detect genetic locus by genetic analyzer(ABI 3730XL).

1 Experimental result

Genotype Test Results

**Sample NO.**	**Multiple Alleles**	**Non - human Source Pollution**	**Matched Cell Line**
01	(−)	(−)	ESC

2 STR Typing Result

Marker	STR Profile(**sample 01**)				STR Profile(DSMZ)		
	Allele1	Allele2	Allele3	Allele4	Allele1	Allele2	Allele3
D5S818	8	10			8	10	
	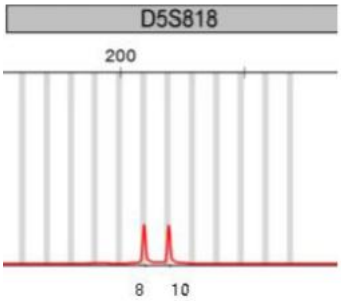						
D13S317	11	11			11	11	
	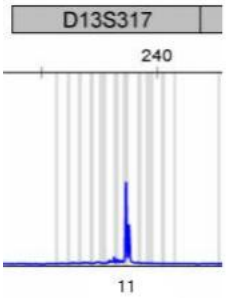						
D7S820	11	11			11	11	

	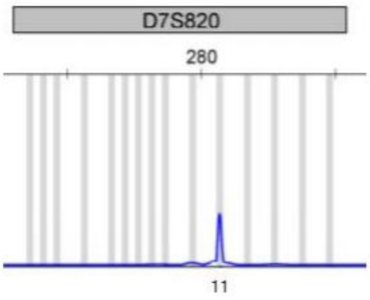						
D16S539	12	13			12	13	
	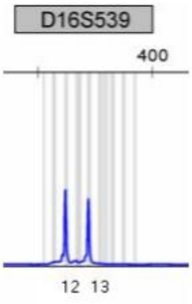						
vWA	14	18			14	18	
	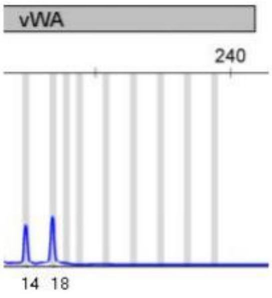						
AMEL	X	X			X	X	
	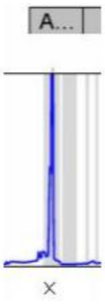						
TPOX	9	9			9	9	
	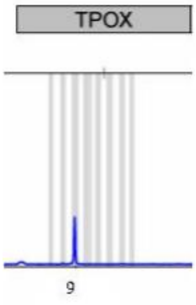						

CSF1PO	11	12			11	12	
	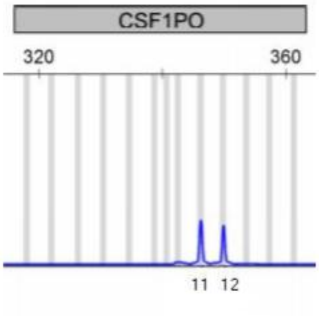						

3 Programme

	Programme1	Programme2	Programme3	Programme4	Programme5	Programme6
**1**	**TH01**	**AMEL**	**TPOX**	**D3S1358**	**PENTAE**	**D19S433**
**2**	**D12S319**	**D5S818**	**vWA**	**D13S317**		**PENTAD**
**3**	**D7S820**	**D2S1338**	**D8S1179**	**D6S1043**		
**4**	**CSF1P0**	**D21S11**		**D16S539**		
**5**	**FGA**	**D18S51**				

Laboratory Technician：Xin Wang

Review Member: Muyi Yang
